# Extraction Optimization of Polysaccharides from Wet Red Microalga *Porphyridium purpureum* Using Response Surface Methodology

**DOI:** 10.3390/md22110498

**Published:** 2024-11-04

**Authors:** Yi Chen, Qianmei Li, Bingqi Xu, Wenzhou Xiang, Aifen Li, Tao Li

**Affiliations:** 1 CAS Key Laboratory of Tropical Marine Bio-Resources and Ecology, Guangdong Key Laboratory of Marine Materia Medica, South China Sea Institute of Oceanology, Chinese Academy of Sciences, Guangzhou 510301, China; chenyi2021301@163.com (Y.C.); xubingqi24@mails.ucas.ac.cn (B.X.); xwz@scsio.ac.cn (W.X.); 2 Institute of Hydrobiology, Jinan University, Guangzhou 510632, China; lqm8523@stu2021.jnu.edu.cn

**Keywords:** *Porphyridium*, intracellular polysaccharides, wet biomass, response surface methodology, microwave-assisted extraction

## Abstract

*Porphyridium* is a unicellular marine microalga that is rich in polysaccharides and has excellent biological activities. Optimizing the extraction of polysaccharides can significantly improve the value of *Porphyridium* biomass. In the present study, response surface methodology was employed to optimize the extraction conditions of polysaccharides, including extraction time, extraction temperature, and biomass-to-water ratio. Furthermore, microwave-assisted extraction was used to improve the yield of polysaccharides further. The results showed that increasing the extraction temperature and extraction time could enhance the yield of polysaccharides. The multiple regression analysis of RSM indicated that the model could be employed to optimize the extraction of polysaccharides. The optimal extraction time, extraction temperature, and biomass-to-water ratio were 45 min, 87 °C, and 1:63 g mL^−1^, respectively. Under these optimal conditions, the maximum yield of polysaccharides was 23.66% DW, which well matched the predicted yield. The results indicated that the extraction temperature was the most significant condition affecting the yield of polysaccharides. The microwave-assisted extraction could further improve the yield of polysaccharides to 25.48% DW. In conclusion, hot water with microwave-assisted extraction was effective for polysaccharide extraction in *P. purpureum*.

## 1. Introduction

Microalga polysaccharides have excellent antioxidant, immunomodulatory, antiviral, hypoglycemic, and hypolipidemic activities [1,2]. For example, the polysaccharides of the red macroalga *Pyropia yezoensis* have the potential to scavenge hydrogen peroxide and alkyl radicals [3]. The polysaccharides from the microalga *Limnospira platensis* (formerly *Spirulina platensis*) were demonstrated to inhibit the replication of the herpes simplex virus 1, mumps virus, and influenza A virus [4].

*Porphyridium purpureum* is a unicellular marine red microalga that can synthesize a variety of high-value bioactive products, including intracellular polysaccharides, exopolysaccharides, polyunsaturated fatty acids, and B-phycoerythrin [5]. The exopolysaccharides of *P. purpureum* have become a research hotspot in recent years. The exopolysaccharides of *P. purpureum* have potential applications in functional foods, pharmaceuticals, and skin care cosmetics [6] due to their antioxidant [7], antiviral [8,9], and immunomodulatory activities [10,11]. However, to date, the large-scale production of exopolysaccharides has not been achieved because of the low yield and difficulty in harvesting (the high viscosity of exopolysaccharides makes it difficult to separate from the cells). The content of intracellular polysaccharides of *P. purpureum* can reach 15−52% DW (dry weight) [12], and it consists of glucuronic acid and several neutral monosaccharides (xylose, galactose, and glucose) [13]. The yield of intracellular polysaccharides of *P. purpureum* is 2.19 g L^−1^, which is much higher than the yield of exopolysaccharides (0.34 g L^−1^) [12]. Moreover, the composition of intracellular polysaccharides is similar to that of exopolysaccharides [14], and these polysaccharides may also have bioactivities and applications similar to those of exopolysaccharides. However, there are few studies on the extraction of intracellular polysaccharides of *P. purpureum*.

Conventional extraction methods for polysaccharides include hot water extraction, acid extraction, and alkali extraction [15]. Recently, several novel methods for polysaccharide extraction, such as microwave-assisted, ultrasound-assisted, enzyme-assisted, and pressurized liquid extraction, have been used to improve extraction efficiency and reduce energy consumption [16,17,18,19]. For conventional extraction methods for polysaccharides, temperature, time, and biomass-to-water ratio have great effects on the extraction efficiency. The extraction yield of alginate from *Sargassum binderi* is affected by temperature. The maximum yield of alginate is 27% DW at 90 °C [17]. Response surface methodology (RSM) is an ideal approach to avoid the interaction of a single extraction condition [20]. By RSM, the optimal extraction conditions for *Fucus vesiculosus* polysaccharides are 120 psi for the pressure, 1 min for the extraction time, and 1:25 g mL^−1^ for the biomass-to-water ratio [21]. Similarly, the optimal extraction conditions for the polysaccharides of *Isocrysis galbana* (Haptophyta) are 3.6 h for the extraction time, 67 °C for the extraction temperature, and 1:9 g mL^−1^ for the biomass-to-water ratio [22].

Water as an extractant can preserve the structural information of polysaccharides and reduce the damage to their activity. Microwave-assisted extraction (MAE) directly generates heat through ion conduction and dipole rotation [16], which has been widely used to improve the extraction efficiency of natural products. In the present study, the extraction method of *P. purpureum* polysaccharides is investigated by RSM and MAE. The results will provide an optimal strategy to obtain high-efficiency polysaccharide extraction from *P. purpureum*.

## 2. Results

### 2.1. Effects of Temperature, Time, and Biomass-to-Water Ratio on the Extraction Yield of Polysaccharides

**Figure 1:**
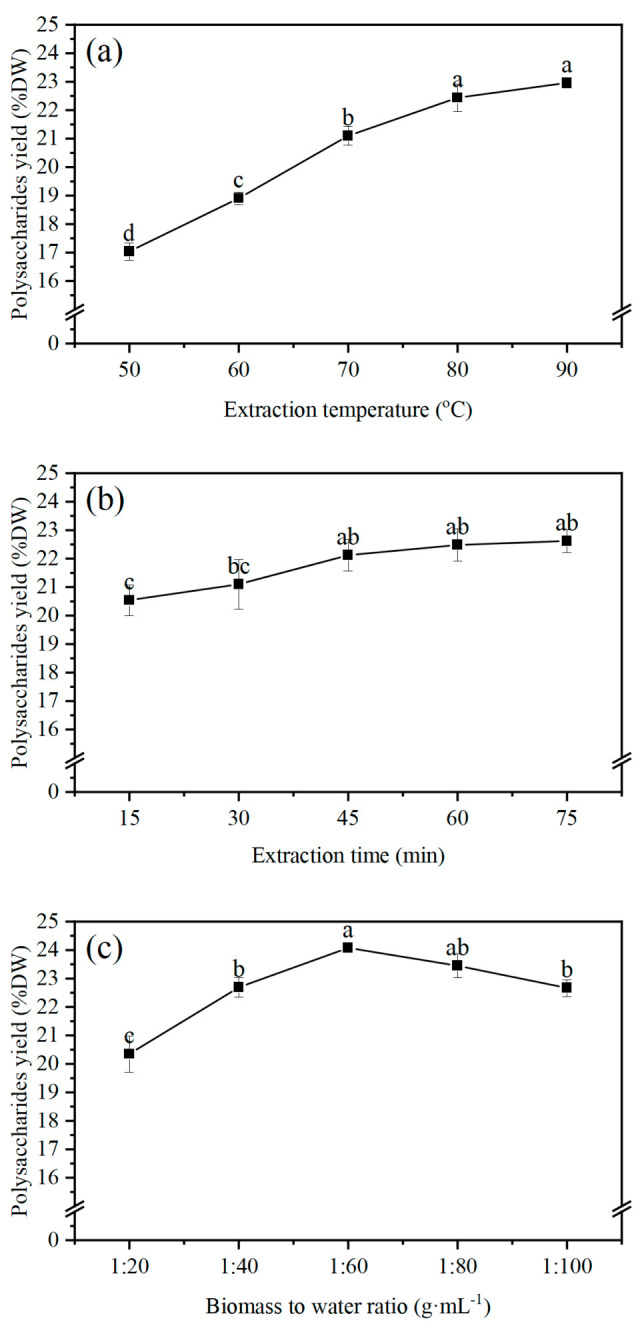
The effects of extraction temperature, extraction time, and biomass-to-water ratio on the yield of polysaccharides: (**a**) extraction temperature (the extraction time and biomass-to-water ratio were fixed at 60 min and 1:50 g mL^−1^); (**b**) extraction time (the extraction temperature and biomass-to-water ratio were fixed at 80 °C and 1:50 g mL^−1^); (**c**) biomass-to-water ratio (the extraction temperature and extraction time were fixed at 80 °C and 60 min). Each mean value represents the mean value of three biological replicates and three technical replicates, *n* = 6. The error bars represent the standard deviations of those means, and different letters indicate significant differences among the treatments at the *p* = 0.05 level.

The extraction yields of polysaccharides under different extraction temperatures (50 °C, 60 °C, 70 °C, 80 °C, and 90 °C) are shown in Figure 1a. Within the temperature range of 50−80 °C, there was a positive correlation between the extraction temperature and the yield of polysaccharides. However, there were no significant differences in the yield as the extraction temperature exceeded 80 °C. The maximum yield was 22.96% DW, which was obtained at 90 °C. Based on these results, the optimal extraction temperature was 80 °C.

The extraction yields of polysaccharides under different extraction times (15 min, 30 min, 45 min, 60 min, and 75 min) are shown in Figure 1b. The extraction yield of polysaccharides increased as the time increased from 15 to 45 min, but there were no significant differences in the yield when the extraction time was beyond 45 min. The maximum yield (22.62% DW) was obtained at 75 min. This result indicated that the polysaccharides were mainly released at the initial stage of extraction. Thus, the optimal extraction time was 45 min.

The extraction yields of polysaccharides under different biomass-to-water ratios (1:20, 1:40, 1:60, 1:80, and 1:100 g mL^−1^) are shown in Figure 1c. The yield increased rapidly when the biomass-to-water ratio ranged from 1:20 to 1:60 g mL^−1^; the maximum yield (24.08% DW) was obtained at 1:60 g mL^−1^. However, when the biomass-to-water ratio ranged from 1:60 to 1:100 g mL^−1^, the yield of polysaccharides decreased from 24.08% DW to 22.67% DW. Therefore, the optimal biomass-to-water ratio was 1:60 g mL^−1^.

Based on the results of the single-factor experiment, the optimal extraction conditions were an 80 °C extraction temperature, a 45 min extraction time, and a 1:60 g mL^−1^ biomass-to-water ratio.

### 2.2. Statistical Analysis and Model Fitting Based on RSM

The interaction effects of temperature, time, and biomass-to-water ratio on the extraction yield of polysaccharides were investigated by using multiple regression analysis. There was a total of 17 groups (Table 1). The extraction yields of polysaccharides were analyzed by using the Box–Behnken Design (BBD) method. Based on the results of the multiple regression analysis, the predicted extraction yield could be fitted into the following second-order polynomial equation:

*Y* = 23.27 + 0.42 × A + 1.23 × B + 0.83 × C − 0.11 × A × B + 0.17 × A × C − 0.16 × B × C − 1.84 × A^2^ − 0.97 × B^2^ − 1.30 × C^2^, (1)

where *Y* is the extraction yield of polysaccharides, and A, B, and C are the extraction time, the extraction temperature, and the biomass-to-water ratio, respectively.

The analysis of variance (ANOVA) was used to analyze the significance and suitability of the model. As shown in Table 2, the *F*-value of this model was 53.58 (*p* < 0.05), implying that the model was used to predict the yield of polysaccharides. The lack of fit was insignificant (*p* > 0.05), confirming the validity of the model. The determination coefficient (*R*^2^) was 0.9857, indicating that only 1.43% of the total variation could not be explained by the model. The 0.9673 adjusted *R*^2^ showed that the model was highly significant. At the same time, the coefficient of the variation (C.V.% = 1.47%) further proved the good reliability and precision of the model. The adeq. precision was 17.222, which was higher than the critical value of 4, indicating the accuracy of the model. The regression model indicated that this model was adequate to predict the yield of polysaccharides in the present study.

The regression coefficients of the equation are listed in Table 2. The linear coefficients (A, B, and C) and quadratic coefficients (A2, B2, and C2) had remarkable influences on the polysaccharide yield (*p* < 0.05). The cross-product coefficients (AB, AC, and BC) had no significant influence on the yield of polysaccharides (*p* > 0.05). By analyzing the linear and quadratic coefficients, it could be concluded that the order of extraction conditions influencing the polysaccharide yield, from strong to weak, was extraction temperature, biomass-to-water ratio, and extraction time.

### 2.3. Interaction Among Extraction Conditions

**Figure 2:**
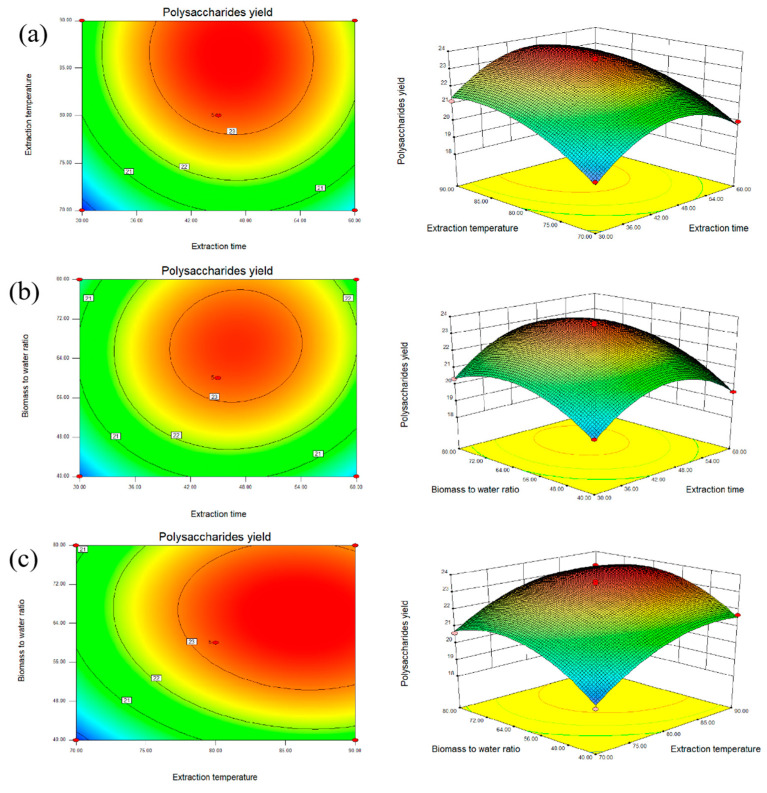
The contour plots and response surface plots of the effects of the extraction temperature, extraction time, and biomass-to-water ratio on the polysaccharide yield: (**a**) the contour plot and response surface plot of the effects of the extraction time, extraction temperature, and their interaction on the extraction yield; (**b**) the contour plot and response surface plot of the effects of the extraction time, biomass-to-water ratio, and their interaction on the extraction yield; and (**c**) the contour plot and response surface plot of the effects of the extraction temperature, biomass-to-water ratio, and their reciprocal interaction on the extraction yield.

Figure 2a shows the interaction effect between the extraction time and the extraction temperature on the yield when the biomass-to-water ratio was fixed at 1:60 g mL^−1^. The 3D plot was steep and the 2D plot was elliptical. The plots indicated that the optimal polysaccharide yield could be obtained when the extraction temperature was in the range of 78 °C–90 °C and the extraction time was in the range of 37–56 min. The extraction temperature showed a synergistic effect on polysaccharide yield when coupled with the extraction time.

Figure 2b shows the interaction effect between the extraction time and the biomass-to-water ratio on the yield when the extraction temperature was fixed at 80 °C. The yield increased quickly to the maximum value as the biomass-to-water ratio ranged from 1:55 to 1:78 g mL^−1^ and as the extraction time ranged from 40 to 54 min. The interaction of the extraction time and the biomass-to-water ratio showed a positive synergistic effect on the yield of polysaccharides.

Figure 2c illustrates the interaction effect between the extraction temperature and the biomass-to-water ratio on the yield when the extraction time was fixed at 45 min. The yield of polysaccharides increased rapidly with the increase in the extraction temperature and the biomass-to-water ratio. The maximum yield was obtained when the extraction temperature ranged from 77 °C to 90 °C and when the biomass-to-water ratio ranged from 1:50 to 1:81 g mL^−1^. The extraction temperature showed a synergistic effect on the yield of polysaccharides when coupled with the biomass-to-water ratio.

By using the software of Design-Expert 8.0.6, the quadratic polynomial equation was solved. The optimal extraction conditions by RSM were that the extraction time was 44.81 min, the extraction temperature was 86.75 °C and the biomass-to-water ratio was 1:62.55 g mL^−1^. The predicted polysaccharide yield under the condition was 23.71% DW. To validate the accuracy of the predicted result, a new experimental group was set up. The extraction conditions were as follows: extraction time of 45 min, extraction temperature of 87 °C, and biomass-to-water ratio of 1:63 g mL^−1^. Under these conditions, the extraction yield of polysaccharides reached 23.66% DW. There were no significant differences between the predicted yield and the experimental yield (*p* > 0.05), indicating that the model equation was reliable and accurate.

### 2.4. Effect of Microwave-Assisted Extraction on the Yield of Polysaccharides

**Figure 3:**
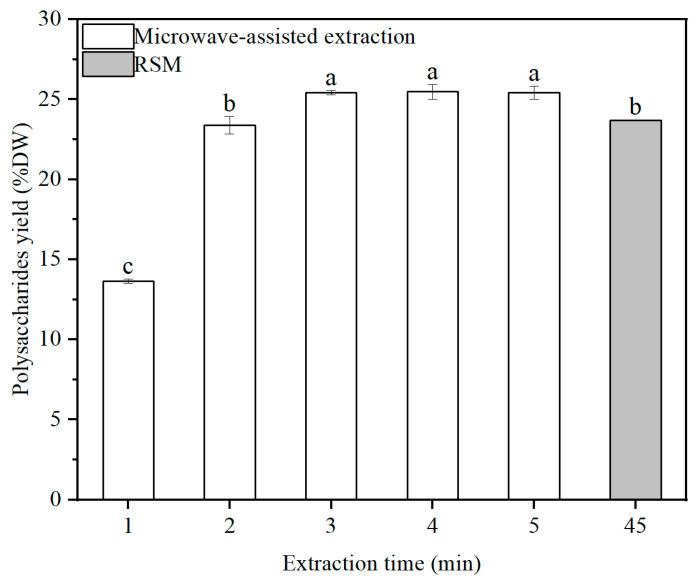
The yields of polysaccharides obtained by microwave-assisted extraction and response surface methodology. The error bars represent standard deviations from three independent samples. The different letters (a–c) indicate significant differences among the treatments at the *p* = 0.05 level.

As shown in Figure 3, the extraction yield of polysaccharides showed an increasing trend as the extraction time ranged from 1 to 5 min by using microwave-assisted extraction. The yield of polysaccharides increased from 13.63% DW to 25.41% DW when the extraction time increased from 1 to 3 min. There was no significant difference in the yield of polysaccharides at 3−5 min (*p* > 0.05). The maximum yield of polysaccharides was 25.48% DW at 5 min. At the same time, the extraction efficiency of polysaccharides reached 96.66%. Moreover, the yield of polysaccharides obtained by microwave-assisted extraction was 7.69% higher than the yield of polysaccharides obtained by RSM extraction (23.66% DW).

## 3. Discussion

The polysaccharides of *P. purpureum* are widely available with the potential for application in medicines, foods, and cosmetics [5]. The content of polysaccharides of *P. purpureum* can reach 52% DW [12]. In our previous study, the extraction yield and extraction efficiency of polysaccharides were found to be 22.43% DW and 85.12% by hot water extraction with an extraction time of 240 min (unpublished). It is necessary to find an efficient extraction method to improve the yield of polysaccharides.

Increasing the extraction temperature can enhance the solubility of polysaccharides and promote the release of polysaccharides into the water [23]. The present study showed that the yield of polysaccharides increased from 17.04% DW to 22.96% DW as the extraction temperature increased from 50 °C to 90 °C (Figure 1a). Similarly, the polysaccharide yield of *Ulva intestinalis* (Chlorophyta) increased from 2.7% DW to 8.0% DW as the temperature increased from 30 °C to 90 °C [24]. The polysaccharide yield of *Isocrysis galbana* increased significantly with the increase in temperature [22]. The reason might be that the high temperature improved the solubility and diffusion rate of polysaccharides [25]. The polysaccharides extraction at room temperature is more favorable for large-scale industrial production as it can significantly reduce energy consumption. The enzyme-assisted extraction of polysaccharides is often carried out at room temperature. The high-efficiency commercial enzyme is essential for enzyme-assisted extraction. If the activity of the existing commercial enzyme is low, the time-consuming screening and modification of the enzyme will have to be conducted. In contrast, the heating method in the present study is commonly used for polysaccharide extraction. In addition, the cells of *P. purpureum* are encapsulated by a thick gelatinous layer, which limits the release of intracellular polysaccharides [5]. High-temperature extraction can effectively destroy the gelatinous layer to accelerate the release of polysaccharides.

Extraction time is also an important factor that affects extraction efficiency. In this study, the yield of polysaccharides showed an increasing trend with the extraction time increased from 15 min to 45 min (Figure 1b). This finding indicated that the polysaccharides were mainly released during the initial stage of extraction. In addition, there was no significant difference in the yield of polysaccharides after 45 min, probably because the temporal requirement for the polysaccharides to be released into the solvent was met at this time. The maximum yield of polysaccharides reached 22.62% DW, and the extraction time was 3−5 times shorter than that of the hot water extraction method we used before. The yield of polysaccharides of *Chlorella* sp. increased rapidly as the extraction time increased within 3 h [26]. The polysaccharide yield of *L. platensis* increased from 8.2% DW to 12.2% DW as the extraction time increased from 5 to 35 min [27].

The concentration difference between the inside and outside of the cell increases the diffusion of polysaccharides [28]. The present study showed that the polysaccharide yield of *P. purpureum* enhanced rapidly with the increase in the biomass-to-water ratio (Figure 1c). The highest yield (24.08% DW) was obtained at 1:60 g mL^−1^. This was consistent with the results of Song et al. [26], who reported that the polysaccharide yield of *Chlorella* sp. increased from 3.57% to 7.38% as the ratio increased from 1:10 to 1:40 mg mL^−1^. The maximum yield (3.05% DW) of *Dictyopteris divaricata* was obtained at 1:110 g mL^−1^ [29]. The reason might be that the low concentration of polysaccharides had a low viscosity in the solution, thus improving the solubilization and diffusion of polysaccharides [30]. However, as the biomass-to-water ratio increased from 1:60−1:100 g mL^−1^, the yield of polysaccharides showed a decreasing trend. It could be inferred that the high biomass-to-water ratio extended the diffusion distance, resulting in a decrease in the polysaccharide yield. Similar results were obtained by Rahimi et al.; the polysaccharide yield of *Ulva intestinali* (Chlorophyta) showed a decreasing trend when the biomass-to-water ratio exceeded 1:60 g mL^−1^ [24]. Moreover, the polysaccharide yield of *Dictyosphaerium* sp. showed an upward trend as the ratio increased and the maximum polysaccharide yield was 6.82% DW. However, when the biomass-to-water ratio exceeded 1:25 g mL^−1^, the yield showed a decreasing trend [31]. Therefore, to obtain a high polysaccharide yield, it is essential to determine the optimal range of biomass-to-water ratios.

The use of an ethanol/water solvent mixture is common in food extraction. However, the ethanol/water solvent mixture is not suitable for extracting polysaccharides. In order to improve the extraction yield of polysaccharides from *P. purpureum*, we used the ethanol/water solvent mixture to extract polysaccharides. The results showed that the extraction yield of polysaccharides did not significantly increase. Unfortunately, using this solvent mixture as an extractant changed the color of polysaccharides to dark green, which could reduce the quality of polysaccharides. The reason for this phenomenon might be that the ethanol/water solvent mixture could extract a small amount of chlorophyll and carotenoids, which are lipid-soluble compounds. Furthermore, considering the large-scale production of polysaccharides, the application of ethanol will increase the safety level and the costs of the enterprise. In our study, the in-situ structural information of polysaccharides can be preserved by using water as an extractant. Additionally, polysaccharides are insoluble in ethanol, and ethanol is commonly used to harvest polysaccharides from extracts.

RSM is an effective method to reduce the costs, energy, and time in the process of extraction [20]. It is widely used in optimizing the extraction conditions, such as polysaccharides, vitamin E, anthocyanins, and phycobiliprotein [31,32,33]. In the present study, the optimal extraction conditions for the polysaccharides of *P. purpureum* by RSM were an extraction time of 45 min, extraction temperature of 87 °C, and biomass-to-water ratio of 1:63 g mL^−1^. In addition, the extraction yield of polysaccharides (23.66% DW) using RSM was significantly higher than the yield using hot water extraction (22.43% DW). Moreover, the extraction time was 4 times shorter compared to the hot water extraction methodused in our previous study. By RSM, the optimum extraction conditions for the polysaccharides of *Dictyopteris divaricata* were determined to be a biomass-to-water ratio of 1:110 g mL^−1^, an extraction time of 6 h, and an extraction temperature of 100 °C [29]. Similarly, the optimal extraction conditions for the polysaccharides of *Caulerpa lentillifera* were a biomass-to-water ratio of 1:10 g mL^−1^, an extraction temperature of 90 °C, and an extraction time of 45 min [23]. The polysaccharide yield (9.29% DW) of *Rhodosorus* sp. was markedly higher than that by hot water extraction, with an extraction yield of 3.13% DW [34]. Therefore, RSM is usually used to identify the optimum extraction conditions. The main advantage of RSM is the reduced number of experimental groups needed to evaluate multiple extraction conditions and their interactions [20]. RSM is more efficient and easier to use to arrange experiments.

MAE can improve the extraction yield of polysaccharides while reducing the solvent and energy consumption levels [16]. In the present study, the polysaccharide yield increased obviously at 1−3 min. Heat is generated directly into the cell matrix by the permeation of electromagnetic energy, leading to the acceleration of polysaccharide transfer [35]. The results showed that the yield of polysaccharides by microwave-assisted extraction had no significant differences after 3 min, and the maximum yield (25.48% DW) was higher than that resulting from hot water extraction (22.43% DW) in our previous study. Similarly, the extraction yield of polysaccharides from *Ulva meridionalis* using MAE was 40.40% DW, which was 21.7% greater than that of the conventional method [16]. In addition, the yield of fucoidan extracted from *Fucus vesiculosus* by MAE increased 17-fold compared to the conventional method [21]. The reason might be that the energy absorption led to the redistribution of energy between the molecules, which caused penetration of the solvent into the substrate.

The physical and chemical properties of polysaccharides are crucial for commercial applications. Polysaccharides obtained by different extraction methods may have different physical properties and activities. For example, different extraction temperatures lead to different monosaccharide compositions of the macroalga *Ascophyllum nodosum*. Glucuronic acid is the major component of polysaccharides extracted at a temperature of 150 °C, while fucose is the major component of polysaccharides extracted at 90 °C [16]. Based on the research purposes of different products in the future, our study will be conducted on the effects of extraction conditions on the physical properties and activities of the polysaccharides, such as antioxidant activity for functional foods and moisturizing activity for cosmetics. This is also a complex and systematic study.

In the present study, the extraction material was wet biomass. The drying of wet biomass usually consumes a large amount of energy, which accounts for 40% of the costs. The results of this study showed that polysaccharides could be directly extracted from wet biomass. Moreover, the extraction efficiency was more than 90%, providing a reference solution for the commercial application of *P. purpureum* in the future. Even though microwave-assisted extraction further improved the yield of polysaccharides and reduced the extraction time, it required additional consumption costs and corresponding equipment [36]. Its application in marine polysaccharide extraction has so far been limited to laboratory research since few studies on an industrial scale have been reported. If MAE is used to promote the large-scale industrial production of polysaccharides, it is necessary to find a balance between polysaccharide value and consumption. In addition, the development of the standard extraction method is crucial to protect the structural integrity and bioactivities of polysaccharides [37]. However, the excessive extraction temperature and microwave power might cause the degradation of polysaccharides themselves and damage their structures [2]. Therefore, it is important to develop a simple and reliable structural characterization for polysaccharides, which will contribute to a better understanding of the structure–bioactivity relationship in polysaccharides.

## 4. Materials and Methods

### 4.1. Microorganisms and Culture Conditions

*P. purpureum* FACHB 806 was purchased from the Freshwater Algae Culture Collection of the Institute of Hydrobiology in China. *P. purpureum* FACHB 806 were cultured in the modified ASW medium [12]. Illumination was provided by T8 fluorescent lamps (Philips; Suzhou, China) at 300 µmol photons m^–2^ s^–1^. The photoperiod was 24 h: 0 h (light: dark). The culture temperature was 25 ± 1 °C. CO_2_-enriched compressed air (1% CO_2_, v:v) was continuously bubbled into the photobioreactor to provide a carbon source. After 10 days of cultivation, the culture was centrifuged at 8000 rpm for 10 min and washed with deionized water three times. The wet biomass was collected and stored at −20 °C.

### 4.2. Determination of the Content of Polysaccharides and Moisture of Wet Biomass

Moisture content of wet biomass: Wet biomass (100 mg) was added to pre-weighed filters. The filters were dried at 80 °C to a constant weight and re-weighed.

W = (m_2_ − m_1_)/100 × 100%, (2)

where W is the moisture content of wet biomass, m_1_ (mg) is the weight of the pre-weighed filter, and m_2_ (mg) is the weight of the filter with dry biomass. The moisture content of the wet biomass used in this study was determined to be 90%.

Polysaccharide content: The wet biomass was freeze-dried using an FD-1-50 freeze dryer (Boyikang, Beijing, China). The freeze-dried biomass was used to measure the polysaccharide content. The freeze-dried biomass (m_1_) was hydrolyzed with 1.0 N H_2_SO_4_ at 80 °C for 1 h. The supernatant was collected by centrifugation at 8000 rpm for 5 min, and this process was repeated three times. The concentration of polysaccharides in the supernatant was determined according to the phenol–sulfuric acid method [38]. The polysaccharide content was estimated using the following equation:

Polysaccharide concentration (% DW) = C × V/m_1_ × 100%, (3)

where C (mg mL^−1^) is the concentration of polysaccharides, V (mL) is the total volume of the supernatants, and m_1_ (mg) is the weight of the freeze-dried biomass.

### 4.3. Single-Factor Experiment

Wet biomass (100 mg) was added to a glass centrifuge tube. The single extraction condition included the extraction temperature (50 °C, 60 °C, 70 °C, 80 °C, and 90 °C), the biomass-to-water ratio (1:20, 1:40, 1:60, 1:80, and 1:100 g mL^−1^) and the extraction time (15 min, 30 min, 45 min, 60 min, and 75 min). The extracts were collected by centrifugation at 4000 rpm for 5 min, and the supernatants were combined; this process was repeated three times. The polysaccharide content was determined according to the phenol–sulfuric acid method [38]. The polysaccharide yield was estimated using the following equations:

Polysaccharide yield (% DW) = C × V/100× (1 − W) × 100%(4)

where C (mg mL^−1^) is the concentration of polysaccharides, V (mL) is the total volume of the supernatants, and W (%) is the moisture content of wet biomass.

### 4.4. RSM Experiment

Wet biomass (100 mg) was added to a glass centrifuge tube. Based on the results of the single-factor experiment, the Box–Behnken Design (BBD) was used to set the extraction conditions of the RSM experiment. The factors and levels are shown in Table 3. After the extraction process was completed, the extracts were collected by centrifugation at 4000 rpm for 5 min, and the supernatants were combined; this process was repeated three times. The polysaccharide content was determined according to the phenol–sulfuric acid method [38]. The extraction yield of polysaccharides was used as the dependent variable. The whole RSM experiment contains 17 experimental groups (Table 1). The interactions among the extraction conditions and their corresponding optimum levels were expressed by a second-order polynomial equation. The calculation method of polysaccharide yield refers to 4.3.

### 4.5. Microwave-Assisted Extraction

Wet biomass (100 mg) was added to a glass centrifuge tube. The microwave power was 210 W using the M1-L213B microwave (Guangdong Midea Electric Appliances Co., Ltd., Foshan, China). The different extraction times (1−5 min) were set to evaluate the yield of polysaccharides. After the extraction was completed, the extracts were collected by centrifugation at 4000 rpm for 5 min, and the supernatants were combined. This process was repeated three times. The polysaccharide contents of different extraction methods were determined according to the phenol–sulfuric acid method [38]. The calculation method of the polysaccharide yield refers to Section 4.3.

### 4.6. Statistical Analysis

All the treatments had three independent biological replicates and three technical replicates. Multiple regression analysis of RSM was carried out using Design-Expert Software (8.0.6). One-way analysis of variance (ANOVA) was performed to determine the significant differences between the target datasets using SPSS version 18.0 software (SPSS, Chicago, IL, USA). Differences between sample means were analyzed using the least significant difference method (significance level, *p* < 0.05).

## 5. Conclusions

In summary, the results showed that the increasing extraction temperature and extraction time could enhance the yield of polysaccharides. The optimal extraction method by RSM was an effective method to obtain polysaccharides from *P. purpureum*. This method required shorter extraction times and reduced costs compared to the hot water extraction method. The optimal extraction conditions were an extraction time of 45 min, extraction temperature of 87 °C, and biomass-to-water ratio of 1:63 g mL^−1^. Under these conditions, the maximum polysaccharide yield was 23.66% DW, which well matched the predicted yield. Microwave-assisted extraction could further improve the yield of polysaccharides (25.48% DW). Therefore, it was essential to determine the optimal range of the extraction conditions to improve the yield of polysaccharides.

## Figures and Tables

**Table 1:** The response surface design and extraction yield of polysaccharides.

Run	(A) Extraction Time (min)	(B) Extraction Temperature (°C)	(C) Biomass to Water Ratio (g mL^−1^)	Extraction Yield (%DW)
1	45	90	40	21.69
2	60	70	60	19.96
3	30	90	60	21.15
4	45	80	60	23.64
5	60	80	40	19.56
6	45	70	40	18.55
7	45	80	60	23.15
8	30	80	80	20.35
9	60	90	60	21.84
10	30	70	60	18.84
11	45	80	60	23.11
12	60	80	80	21.45
13	45	70	80	20.60
14	45	80	60	23.52
15	45	90	80	23.12
16	45	80	60	22.88
17	30	80	40	19.12

**Table 2:** The ANOVA of the experimental results of the RSM-BBD.

Source	Sum of Squares	df	Mean Square	*F*-Value	*p*-Value
Model	47.28	9	5.25	53.58	<0.0001 **
A	1.4	1	1.4	14.31	0.0069 **
B	12.13	1	12.13	123.68	<0.0001 **
C	5.45	1	5.45	55.53	0.0001 **
AB	0.046	1	0.046	0.47	0.5144
AC	0.11	1	0.11	1.11	0.3270
BC	0.096	1	0.096	0.98	0.3552
A^2^	14.27	1	14.27	145.57	<0.0001 **
B^2^	3.97	1	3.97	40.5	0.0004 **
C^2^	7.1	1	7.1	72.43	<0.0001 **
Residual	0.69	7	0.098		
Lack of fit	0.3	3	0.098	1.01	0.4764
Pure error	0.39	4	0.098		
Cor total	47.97	16			
*R* ^2^	0.9857		C.V.%	1.47	
*R* ^2^_Adj_	0.9673		Adeq. precision	19.028	

** highly significant (*p* < 0.01).

**Table 3:** The independent variables and levels of the response surface method.

Independent Variables	Factor Levels		
	−1	0	1
A (Extraction time, min)	30	45	60
B (Extraction temperature, °C)	70	80	90
C (Biomass-to-water ratio, g mL^−1^)	40	60	80

## Data Availability

The data presented in this study are available from the corresponding author upon request.
